# Communicative roots of complex sociality and cognition: preface to the theme issue

**DOI:** 10.1098/rstb.2022.0115

**Published:** 2022-09-26

**Authors:** Sam G. B. Roberts, Robin I. M. Dunbar, Anna I. Roberts

**Affiliations:** ^1^ School of Psychology, Liverpool John Moores University, Liverpool L3 3AF, UK; ^2^ Department of Experimental Psychology, University of Oxford, Oxford OX2 6GG, UK; ^3^ Institute of Human Biology and Evolution, Adam Mickiewicz University, Poznan, Poland

**Keywords:** communication, cognition, social bonds, primates, vocalizations, gestures

## Abstract

Primates live in stable social groups in which they form differentiated relationships with group members and use a range of communication including facial expressions, vocalizations and gestures. However, how these different types of communication are used to regulate social interactions, and what cognitive skills underpin this communication, is still unclear. The aim of this special issue is to examine the types of cognitive skills underpinning the flexible and complex communication that is used to maintain the bonded social relationships found in primates and humans.

This article is part of the theme issue ‘Cognition, communication and social bonds in primates’.

## 1. Introduction

Imagine an adult female chimpanzee with a young infant living in a community of 60 other chimpanzees in the wild. What are the key challenges she faces? Clearly, she faces ecological challenges involving finding patchily distributed food resources and avoiding predation for her and her offspring [1]. She also faces a multitude of challenges arising from living in a stable, multi-male, multi-female group [2]. She must coordinate her activities (sleeping, resting, moving, feeding) with other group members, to benefit from the protection that group living offers in terms of reducing predation risk [3]. She needs to be able to recognize the other chimpanzees in her community and retain information about their status (e.g. sex, dominance rank) [4]. As chimpanzees live in a fission–fusion system where small parties of chimpanzees forage separately from the rest of the community, she needs to make decisions about which parties to join and thus which other chimpanzees to maintain close proximity to [5]. As a signaller, she uses a variety of communication (facial expressions, gestures, vocalizations) both to coordinate her behaviour with others and to influence conspecifics' behaviour to her own advantage [6–9]. As a receiver of communication, she needs to decide where to focus her attention among the wide variety of communication that may be taking place within her party [10]. If she determines that communication is directed at her, or relevant to her, she needs to integrate cues relating to the signal itself and also contextual information relating to the characteristics of the signaller, the audience and wider ecological context in order to predict conspecifics' behaviour [10,11]. She then needs to choose from a wide set of action opportunities in deciding how to react to the communication [12]. Finally, she needs to maintain both strong social bonds with a small number of chimpanzees to buffer her against the stresses of group living, while also maintaining weaker social bonds with the wider community of chimpanzees [13]. Thus, both communication and cognitive skills are central in enabling primates to meet the challenges of group living [2,10].

Anthropoid primates differ from many other mammal species in living in stable social groups where members form long-term social relationships outside the contexts of mating and rearing offspring [14]. Group living in primates brings benefits in terms of reduction of predation risks but also costs in terms of competing with group mates for food, mates and social partners [15]. Group members form long-term social relationships with conspecifics with both competitive and cooperative elements as different individuals in the group have different energetic needs and genetic interests, and primates with stronger social bonds have better fitness outcomes [16,17]. The cognitive challenges of group living have been proposed as one of the key drivers of brain evolution in primates, with relative brain size associated with group size and other measures of social complexity [18]. Further, the challenges of group living mean that communication plays a central role in social life, as it allows signallers to influence the behaviour of others and receivers to predict signallers’ behaviour [10,19]. Primates flexibly use a range of signals including vocalizations, facial expressions and gestures that show a high degree of voluntary control and these signals play an important role in maintaining and coordinating interactions between group members [6,7,10,19]. The social complexity hypothesis for communication proposes that the demands of living in complex social groups leads to selection pressure for more complex social communication [20]. In this context, social complexity is defined as social networks in which individuals interact with many different individuals across many social contexts, while communicative complexity is defined as systems that contain a larger number of functionally distinct elements, or in which a large number of bits are contained within the signals [20].

However, the specific ways in which primates use different of communication to meet the demands of group living, and what cognitive skills underpin these different types of communication, is still unclear [10,21–23]. While the overall repertoire of signals appears to be highly conserved for vocalizations, gestures and facial expressions, there is flexibility in the production of these signals according to the social and ecological context across all modalities of communication [7,10,19,24]. Thus signallers need to choose which signal to produce in which context in order to achieve their communicative goals and influence the behaviour of the recipient [10,11,19]. Further, the cognitive demands of communication for the receiver have been increasingly recognized, as receivers have to choose which signals to pay attention to, and interpret the signal based on contextual cues including the identity of the signaller (e.g. age and sex), the nature of the signal (e.g. type of vocalization or gesture) and the wider social context (e.g. other primates in close proximity) [8,10,11,19,21]. The aim of this special issue is to examine the types of cognitive skills underpinning the flexible and complex communication that is used to maintain the bonded social relationships found in primates and humans.

The first two papers of this issue describe the key cognitive mechanisms underpinning the processing of social information in primates and how these are related to social and ecological factors. Roberts *et al*. [25] highlight the fact that the chronic and acute stresses caused by group living have a detrimental effect on the very cognitive skills needed to process information about social bonds and goal-directed communication. Intentional communication may play an important role in enhancing cognitive processing in recipients when exposed to stressors by upregulating the dopamine system. Shultz & Dunbar [26] use a meta-analysis to identify separate cognitive and energetic factors associated with brain size in primates, finding that socioecological complexity is associated with absolute brain size and group size, while energetic constraints are associated with relative brain size. Living in large stable groups requires specialized cognitive skills including inhibiting prepotent actions, and, as the range of social and technical behaviours covary, this suggests selection for an increasingly flexible, domain-general cognitive capacity in primates.

The next two papers examine associations between social and communicative complexity across a range of primate species. Fichtel & Kappeler [27] use a phylogenetic analysis of lemurs to show that repertoire sizes in vocal, olfactory and visual modalities are positively associated with group size, but not environmental factors, arguing that communicative complexity in lemurs changed in response to evolutionary changes in social complexity. Aureli *et al*. [28] focus on spider monkeys and highlight some of the challenges of accurately measuring social, communicative and cognitive complexity within and between species. They argue that a more elaborated communicative repertoire may be needed to manage differentiated social relationships in species with fission–fusion dynamics because these present specific cognitive challenges, including keeping track of relationships with absent group members.

Some of the strongest evidence of intentionality in communication across the whole communicative repertoire comes from studies of gestures in great apes [7,29,30]. Intentional communication is characterized by flexibility in the production of a signal to achieve a communicative goal, including sensitivity to the recipients' orientation, response waiting and persisting in communication until the goal of communication is met [22,31]. All great ape species gesture intentionally, but it is less clear how this intentional communication helps meet the social challenges of group living, or what cognitive skills are needed by both signallers and receivers of intentional communication. The next set of articles explore how intentional gestural communication in great apes is related to sociality, the cognitive skills underpinning this communication, and the implications for language evolution. Hobaiter *et al*. [32] highlight similarities between words and gestural communication and set out future directions of study in this area, including how contextual cues are used to interpret gestures, how second-order intentionality can be established, and how gestures are used in back-and-forth interactions. Amici & Liebal [33] focus on how the complexity and effectiveness of gestural communication is associated with individual and dyadic sociality across chimpanzees, orangutans and siamangs. They find that dyads that have stronger social bonds use a larger number of gesture types and that all species flexibly adjust their communication according to the attention of the recipient, supporting the link between social and communicative complexity across both great and lesser apes. Roberts & Roberts [34] examine flexibility in gestural communication in wild chimpanzees, demonstrating signallers used intentional gestures more frequently to recipients who are stressed and these gestures are more likely to evoke approach behaviour by the recipients. This suggests an important role of intentional communication in facilitating coordination of behaviour and the understanding of intentions between signallers and recipients in conditions of stress, such as the presence of a dominant bystander. Finally, Damjanovic *et al*. [35] show that gestural communication, but not vocal or bimodal communication, is associated with a larger social network size in wild chimpanzees and that, with weak social bonds, gestures accompanied by response waiting were more likely to elicit approaches than vocalizations accompanied by elaboration, which elicited avoidance. Overall, this set of papers demonstrate the cognitive complexities involved in intentional gestural communication for both signallers and receivers, and how this communication helps primates to meet the challenges of group living, in terms of maintaining social bonds and coordinating social interactions.

In addition to gestural communication, primates use a range of vocalizations and facial expressions to meet the demands of group living [19,24]. The final set of papers examines how different types of communication are used to manage specific social interactions, and how to measure the level of emotional arousal associated with these interactions. Briseño-Jaramillo *et al*. [36] showed that contact calls in spider monkeys are used flexibly according to the composition of the audience and are used at a higher rate when fission or fusion events take place. Vocalization can be used to obtain information about subgroup members’ locations and identities during fission events, and thus help primates reduce uncertainty and meet the demands of this complex social system. Clarke *et al*. [37] also examine associations between communication and uncertainty and show that a higher level of intensity of facial expressions in wild crested macaques is associated with aggressive as compared to affiliative interactions, especially in interactions between individuals who were closely matched in dominance ratings. This suggests that, in addition to vocalizations and gestures, facial expressions also play an important role in regulating social interactions in primates, although the degree of voluntary control in the production of facial expressions is less clear than for vocalizations or gestures. Heesen *et al*. [38] focus on the flexible use of communication in post-conflict periods in bonobos, showing that the production of paedmorphic signals by victims was sensitive to audience size and composition, increased their chances of receiving consolation and, in adults, reduced the risk of aggression from opponents. This shows how flexible use of a range of vocalizations, facial expressions, gestures and body signals can be used to achieve specific social goals, including reducing the risk of renewed aggression. Finally, a key challenge in this field is measuring the level of arousal associated with different social situations and how this may be associated with patterns of communication. Thermal imaging enables monitoring of physiological states in real time in wild animals and Barrault *et al*. [39] use this technique to show that nasal temperatures were lower when feeding on meat as compared to figs, indicating that social feeding on contested resources is perceived as more stressful. Further, nasal temperatures were affected by the composition of the audience, suggesting chimpanzees monitor their social environment during competitive situations.

Overall the articles in this special issue provide new insights into how communication is used to manage the demands of social life, and the cognitive processes underlying this, including flexible production of signals according to the socio-ecological context by signallers, and detecting and responding to signals by receivers. Several of the contributions also highlighted ongoing challenges in this area of research, including how to measure social, communicative and cognitive complexity within and between species and establishing the cognitive requirements of different types of communication for both signallers and receivers. We hope that this theme issue serves to provide an overview of the key findings in this area and a stimulus for future research.

## Data Availability

This article has no additional data.
